# p53 Immunohistochemistry and Mutation Types Mismatching in High-Grade Serous Ovarian Cancer

**DOI:** 10.3390/diagnostics12030579

**Published:** 2022-02-24

**Authors:** Eunhyang Park, Hyunho Han, Sung-Eun Choi, Hyunjin Park, Ha-Young Woo, Mi Jang, Hyo-Sup Shim, Sohyun Hwang, Haeyoun Kang, Nam-Hoon Cho

**Affiliations:** 1 Department of Pathology, Severance Hospital, Yonsei University College of Medicine, Seoul 03722, Korea; epark54@yuhs.ac (E.P.); path190031@yuhs.ac (H.P.); beliefi@yuhs.ac (H.-Y.W.); shimhs@yuhs.ac (H.-S.S.); 2 Department of Urology, Severance Hospital, Yonsei University College of Medicine, Seoul 03722, Korea; tintal@yuhs.ac; 3 Department of Pathology, CHA Bundang Medical Center, CHA University School of Medicine, Seongnam-si 13496, Korea; sungun86@yuhs.ac (S.-E.C.); blissfulwin@cha.ac.kr (S.H.); 4 Department of Pathology, National Health Insurance Service Ilsan Hospital, Yonsei University College of Medicine, Goyang 10444, Korea; mjrose511@yuhs.ac; 5 Center for Cancer Precision Medicine, CHA Bundang Medical Center, CHA University School of Medicine, Seongnam-si 13496, Korea; 6 Department of Biomedical Science, CHA University, Seongnam-si 13496, Korea

**Keywords:** p53, immunohistochemistry, next generation sequencing, oligomerization domain

## Abstract

High-grade serous carcinoma (HGSCa) of the ovary is featured by *TP53* gene mutation. Missense or nonsense mutation types accompany most cases of HGSCa that correlate well with immunohistochemical (IHC) staining results—an all (missense) or none (nonsense) pattern. However, some IHCs produce subclonal or mosaic patterns from which TP53 mutation types, including the wild type of the gene, cannot be clearly deduced. We analyzed a total of 236 cases of ovarian HGSCa and tumors of other histology by matching the results of p53 IHC staining and targeted next-generation sequencing (TruSight Tumor 170 panel). Ambiguous IHCs that do not belong to the conventional “all or none” groups were reviewed to distinguish the true wild type (WT) from potentially pathogenic subclonal or mosaic patterns. There were about 9% of sequencing-IHC mismatching cases, which were enriched by the p53 c-terminal encoding nuclear localization signal and oligomerization domain, in which the subcellular locations of p53 protein were affected. Indeed, mutations in the oligomerization domain of the p53 protein frequently revealed an unmatched signal or cytosolic staining (L289Ffs*57 (Ins), and R342*). We conclude that both mutation types and IHC patterns of p53 are important sources of information to provide a precise diagnosis of HGSCa.

## 1. Introduction

The tumor suppressor gene *TP53* is one of the most frequently altered genes in human cancers [1]. In particular, the *TP53* mutation rate is highest in high-grade serous ovarian carcinoma (HGSCa), reaching 95% to 100% [2,3]. The most common type of *TP53* mutations in HGSCa is missense mutation (MM, ~70%) which results in p53 protein accumulation in the cell (gain-of-function or dominant-negative), while some occur as nonsense mutation (NM, ~10%) leading to a truncated p53 protein with reduced activity [4]. The p53 protein consists of a transactivation domain, DNA-binding domain, nuclear localization site, and oligomerization domain (From N-terminal to C-terminal). It is possible that mutations occurring in different domains lead to different biological consequences in terms of protein degradation or intracellular location. Most of the *TP53* missense mutations (MM) are found in the DNA binding domain (DBD) encoded by amino acid (AA) residues 102 to 292. In contrast, the transactivation domain, proline-rich domain, or oligomerization domain are enriched with NM or frameshift alterations (40–70% of cases) [5].

Although targeted NGS has become popular with reduced costs and analysis turn-around times, immunohistochemical (IHC) staining remains an accurate and cost-effective method in clinical pathology laboratories. For p53, IHC staining of the p53 antibody is used as a surrogate marker for *TP53* mutations in clinics to diagnose HGSCa. The mutation type of *TP53* can be judged from the IHC results: an all (missense) or none (nonsense) pattern. However, this method is not always accurate, having an unacceptable number of misclassified cases [6,7]. Recent clinical adoption of next-generation sequencing (NGS) has helped identify the mutation type and affected domain of the *TP53* gene in HGSCa, and further provided the status of accompanied genetic alterations. For instance, *BRCA1/2* and *RAD* family mutations frequently co-occur with *TP53* mutation [8,9,10], often featuring a characteristic histology and clinical course providing a therapeutic window for poly-ADP-ribose polymerase (PARP) inhibitors [11].

In this study, we analyze the p53 IHC results’ correlation with the *TP53* gene mutation types by NGS. For cases of IHC-NGS mismatch, we provide our histological viewpoints. More specifically, we focus on how often the commonly used DO7 p53 antibody with an N-terminal epitope (at transactivation domain) reflects both its mutation types (MM or NM) and the affecting functional domain of the protein. We then set up an IHC criteria that can distinguish subclonal heterogeneity or mosaicism from true wild-type staining of the p53 protein. Lastly, we analyze the IHC and NGS mismatching cases and explore other co-existing genetic alterations and affected functional domains.

## 2. Materials and Methods

A total of 236 ovarian carcinoma cases were analyzed by NGS using the oophorectomized formalin-fixed paraffin-embedded (FFPE) samples in the Yonsei University Health System (YUHS, 173 samples) and the Cha Medical Center (CHA, 63 samples) from 2014 to 2019. The histological subtype was reevaluated by all participating genitourinary specialty pathologists, and confirmed by ancillary studies of p53, p16, ER/PR, WT1, HNF1b. All tested cases were reevaluated and validated by p53 immunohistochemistry using a DO7 antibody with N-terminal epitope.

### 2.1. Targeted Next-Generation Sequencing

Targeted DNA and RNA sequencing were performed using TruSight Tumor 170 (Illumina, San Diego, CA, USA). The panel included 151 cancer-related genes with potential single-nucleotide variants (SNVs) and indels, 59 genes with potential amplifications, and 55 genes with fusion and splice variants. Briefly, 40 ng of FFPE tissue-derived DNA and RNA were extracted using the QIAGEN AllPrep DNA/RNA FFPE kit (Qiagen, Hilden, Germany). After hybridization capture-based target enrichment, paired-end sequencing (2 × 150 bp) was performed using a NextSeq sequencer (Illumina) according to the manufacturer’s instructions. Variants with a total depth of at least 100× were included for analysis. Variant interpretation was based on recommendations from the Association for Molecular Pathology, American Society of Clinical Oncology, and College of American Pathologists [12]. Actionable genetic alterations were stratified into one of four levels based on the OncoKB website (http://www.OncoKB.org, (accessed on 1 September 2020). The tier 1 variant included level 1 and level 2 genetic alterations according to FDA-approved biomarkers and standards of care. The tier 2 variant included alterations with compelling clinical or preclinical evidence to drug response.

### 2.2. p53 Immunohistochemistry

Immunohistochemistry for p53 was performed on formalin fixed-paraffin embedded tissue sections using a commercially available mouse monoclonal anti-human antibody (Protein Clone DO-7, cat. #M7001, Dako, CA, USA) at a dilution of 1 in 50. Staining was performed on a representative whole section using an automated staining platform, Ventana (Roche). Heat-Induced Epitope Retrieval (HIER) was performed for 30 min at 97 °C in the manufacturer’s acidic retrieval solution (ER1: VBS part no: AR9961). The percentage of cells showing positive nuclear staining was estimated and reported in four categories: (1) >5% and <30% weakly stained nuclei (wild type—WT), (2) ≥95% positively and strongly stained nuclei (missense type—MM), (3) ≤5% weakly stained nuclei (nonsense type—NM), (4) >30% and <95% positively and strongly stained nuclei (borderline type). Cytoplasmic staining was defined as significant cytoplasmic staining, that is, more than a faint blush, in the presence of variables and less than strong and diffuse nuclear immunoreactivity. Subclonal mutant p53 immunostaining was defined as the combination of more than one pattern of staining (typically WT plus one or more mutant patterns, or two different mutant patterns), with each present in at least 5% of tumor cells [13].

### 2.3. Dataset Visualization and Statistical Analysis

To compare our TP53 NGS results with a public dataset, we downloaded the TGCA ovarian serous cystadenocarcinoma dataset (PanCancer Atlas version) from the cBioPortal.org (https://www.cbioportal.org/ (accessed on 1 September 2020)). OncoPrinter and MutationMapper from the cBioPortal.org were used for dataset visualization and analysis [14,15]. Differences in NGS-IHC results mismatch cases were presented as a table and the chi-square test was performed for the contingency test.

Differences in categorized variables between patients with MM and NM were tested by the chi-square test and Fisher’s exact test. For continuous variables, Student’s *t*-test or Mann–Whitney’s *U* tests were used to compare groups. *P*-values less than 0.05 were considered indicative of statistical significance. All analyses were conducted using IBM SPSS, version 25 for Windows (IBM Corp., Armonk, NY, USA).

## 3. Results

Two hundred and two cases of high-grade serous carcinoma (HGSCa) were used for this analysis. In addition, low-grade serous carcinoma (LGSCa) (*n* = 1), carcinosarcoma (CS) (*n* = 9), endometrioid carcinoma (EMC) (*n* = 9), mucinous carcinoma (MuC) (*n* = 8), clear cell carcinoma (CCC) (*n* = 3), and unclassified metastatic carcinoma (*n* = 4) samples were included for comparative analysis. The mutation types of *TP53* which occurred in the analyzed tumors are presented in Table 1.

*TP53* mutations were discovered in 100% of HGSCa samples and in 95.8% of evaluated non-HGSCa tumors. Four HGSCa cases (2.0%) were classified as a splicing variant. Missense mutations (MMs) occurred mostly at the *TP53* DNA-binding domain (DBD) (98.7%). In contrast, nonsense mutation (NMs) and frameshift mutation occurred less frequently at DBD (62.9% and 66.7%, respectively). All four in-frame mutations occurred at DBD. Three tumors (1.3%) had double mutations, of which the highest allelic frequency was considered in the subsequent analysis.

### 3.1. p53 Immunohistochemistry Results

**Figure 1:**
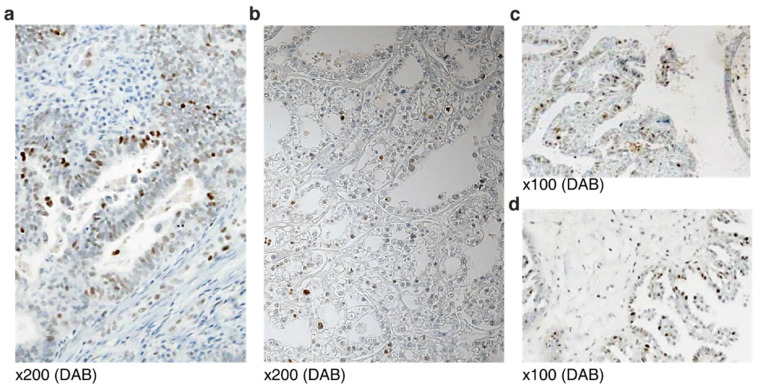
Wild-type p53 protein staining of non-HGSCa ovarian epithelial tumors: (**a**) Endometrioid adenocarcinoma; (**b**) clear cell adenocarcinoma; (**c**) low-grade serous carcinoma; (**d**) micropapillary serous carcinoma.

Wild-type (WT) *TP53* in non-HGSCa samples almost always manifested as scattered weak nuclear staining, seldom exceeding 30% of tumor cells (Figure 1a,b). Endometrioid adenocarcinoma showed simple tubular structures with scattered nuclear staining at the lumen (Figure 1a). Clear cell adenocarcinoma presented with tubule-alveolar structures with characteristic hobnail nuclei of weak staining (Figure 1b). Low-grade serous carcinoma is marked by bullous hierarchical papillary structures with scattered stained cells (Figure 1c). Micropapillary serous carcinoma is characterized by medusa-like projection in bulbous papillae with strong scattered nuclear stainings (Figure 1d).

**Figure 2:**
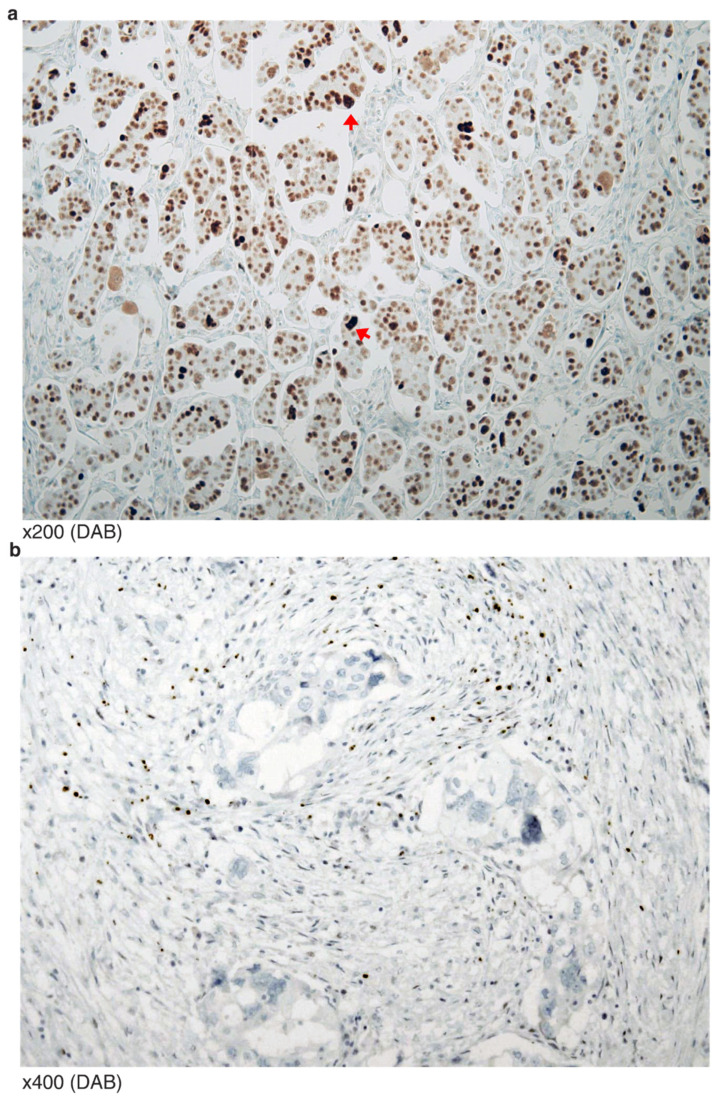
Typical p53 mutation staining of HGSCa: (**a**) All tumor nuclei are strongly positive for p53. Aneuploidy cells are relatively stronger (arrowheads) than the remaining tumor cells; (**b**) no positive stained tumor cell, or “none” pattern.

Typical p53 staining of HGSCa showed “all or none” patterns. The “all” pattern is strong and diffuse nuclear staining of almost all tumor cells (Figure 2a). In this case, aneuploidy tumor cells are often stained stronger than euploidy cells. In the “all” patterned tumors, missense mutation that hinders protein degradation is expected. In contrast, the “none” pattern indicates perfect negative staining (less than 5% weak positive cells) (Figure 2b). In “none” patterned tumors, nonsense or frameshift mutation was expected.

### 3.2. IHC and NGS Data Correlation, Stratified by p53 Functional Domain

**Figure 3:**
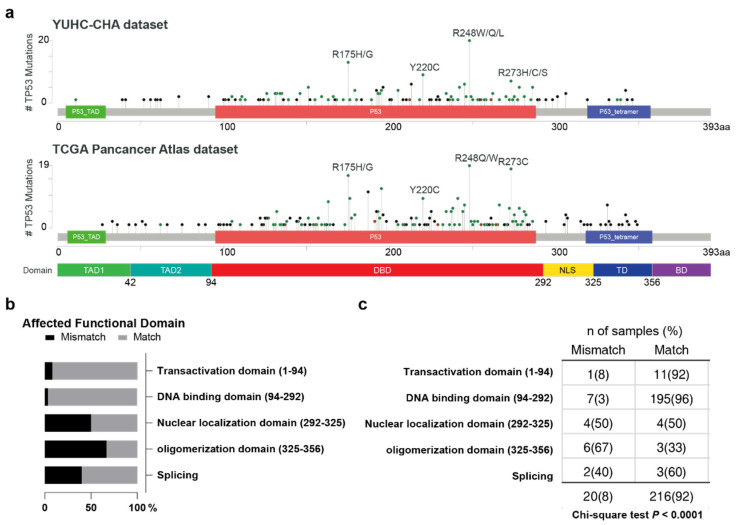
(**a**) Lollipop plot of *TP53* mutation frequencies by their domain site reported in this study and the TCGA ovarian serous cystadenocarcinoma dataset. NLS = nuclear localizing site; OD = oligomerization domain. (**b**,**c**) Distribution of p53 IHC staining and NGS mutation concordance rate. Chi-square test.

Therefore, we analyzed p53 antibody IHC staining results and the corresponding *TP53* mutations in each functional domain (Figure 3). The distribution of TP53 mutations in our dataset (*n* = 236) were comparable to the TCGA ovarian serous cystadenocarcinoma dataset (*n* = 585) (Figure 3a). Interestingly, the rate of IHC staining, or NGS mismatching rate was significantly different across the site of mutations in NGS (Figure 3a,b). The disconcordant cases are described below.

#### 3.2.1. Transactivation Domain (Amino Acid 1–94)

There were 11 *TP53* TAD mutations (seven HGSCa, two endometrioid carcinoma, one mucinous carcinoma and one carcinosarcoma). Eight cases were frameshift deletions, and three cases were NMs, including two W91*. All TAD mutation cases showed a “none” staining pattern, except the endometrioid carcinoma that showed WT-like staining (>30% moderate intensity cells). It had *TP53* frameshift deletion (V73W fs*50).

#### 3.2.2. DNA-Binding Domain (AA 94–292)

Mutations at the *TP53* DNA-binding domain (DBD) were found in 203 cases (175 HGSCa, 8 carcinosarcoma, 7 mucinous carcinomas, 6 endometrioid carcinoma, 3 clear cell carcinoma, 3 metastatic carcinomas, and 1 LGSCa). Among the HGSCa cases, the most frequent mutation type was MM (85.7%), which included R248 (8%), R175 (7.4%) and Y220 (4%). The second most frequent mutation type was NM (11.4%), followed by frameshift deletions (10.9%), frameshift insertions (2.9%) and in-frame insertions (1.7%). These included other previously recognized mutational hotspots—151, 196, 242, 245, 273 and 278 (all 2.9%). IHC staining patterns were mostly concordant with NGS-reported mutation types—170 of 175 HGSCa (97.1%) and 26 of 28 non-HGSCa (92.9%). We describe seven IHC-NGS mismatching cases in DBD (ordered by amino acid sequence).

**Figure 4:**
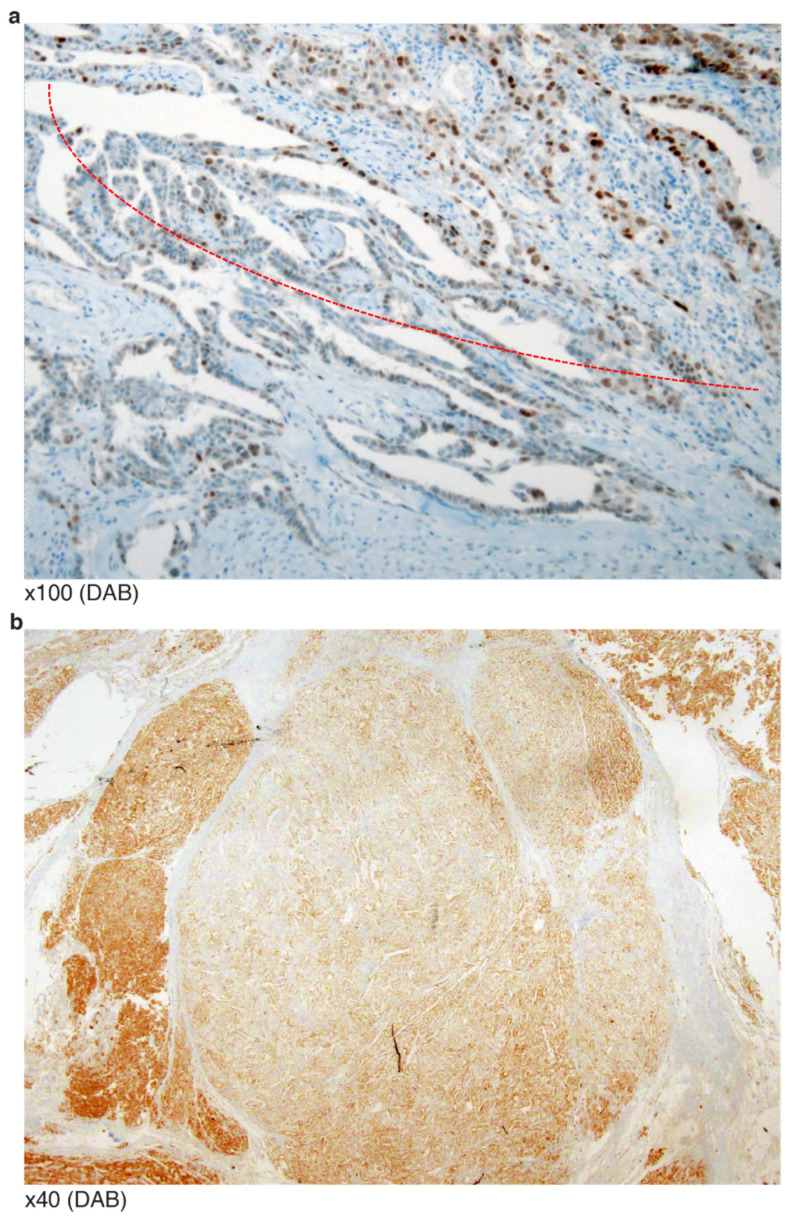
IHC mosaic patterns of p53: (**a**) Partly intense immunoreactivity in the field of papillary serous carcinoma; (**b**) zonal heterogeneity observed in an endometrioid carcinoma.

**Figure 5:**
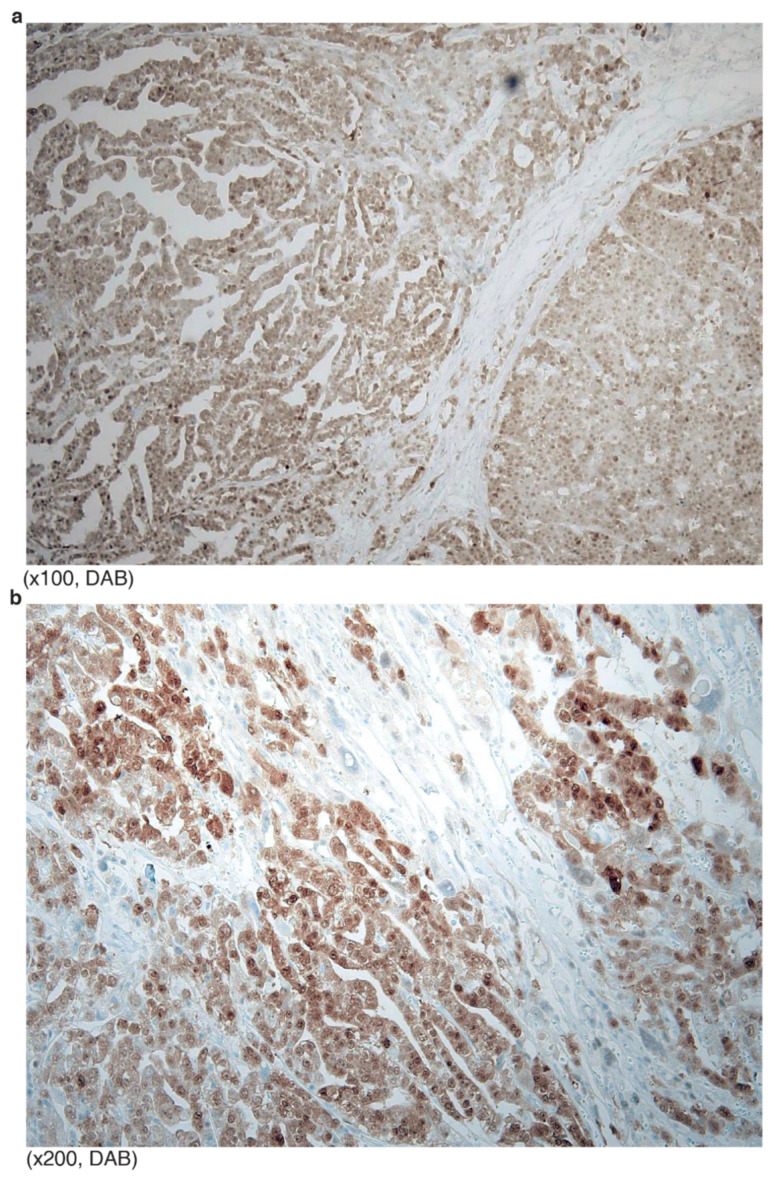
Cytoplasmic staining patterns of p53 from tumors of *TP53* mutations near NLS: (**a**) Heavy signals look like missense mutation, but careful observation revealed cytoplasmic staining (L289F fs*57); (**b**) predominant cytoplasmic staining of p53 (R342*).

1. A clear cell carcinoma of a *TP53* G105R missense mutation (variant allele frequency (VAF) 49.9%) showed a WT-like staining pattern. This was in contrast with another HGSCa of a *TP53* G105V missense mutation, but concordant “all” patterned staining. The clear cell carcinoma case presented with additional *PIK3CA* missense mutation.
2. A HGSCa of probable peritoneal origin was found to have a nonsense mutation of *TP53* (S183*) (VAF 42.7%), but the IHC showed “all” patterned staining. Additional variants were found in *RET*, *RB1* and *ARID1A*, all of unknown significance.
3. A HGSCa presented with a *TP53* in-frame deletion (F212_H214del), but the staining showed diffuse and strong nuclear immunoreactivity. This case has been described in our earlier report [16].
4. An omental HGSCa with extrapelvic peritoneal metastasis revealed a *TP53* G262 in-frame deletion at the end of DBD (VAF 32.9%). IHC showed a mosaic staining pattern (Figure 4a). There was no additional mutation/copy number alteration directly associated with p53 protein degradation.
5. Similar to the above case (G262 in-frame deletion), an endometrioid carcinoma was reported as having a *TP53* S262_G262dup in-frame insertion (VAF 16.7%). However, the staining revealed zonal heterogeneity with some WT-like staining patterns (Figure 4b). In contrast, two HGSCa cases of I254S fs*91 frameshift deletion (VAF 30.3%) and I255del in-frame deletion (VAF 42.1%) presented as having negative or “none” patterned staining. This implies that some non-NMs occurring beyond amino acid 261 of DBD may generate p53 proteins that do not degrade like those with mutations or the earlier amino acid 255 of DBD.
6. A HGSCa presented with a missense mutation of R273H but negative IHC staining, potentially explainable by its low VAF (0.75%).
7. A frameshift insertion at the c-terminal end of DBD (L289F fs*57) presented as complex staining pattens of cytoplasmic retained signals together with heavy nuclear staining of p53 (Figure 5a). It is possible that the mutation may have disrupted the nuclear localization site.

#### 3.2.3. Nuclear Localizing Sites (AA 292–325)

**Figure 6:**
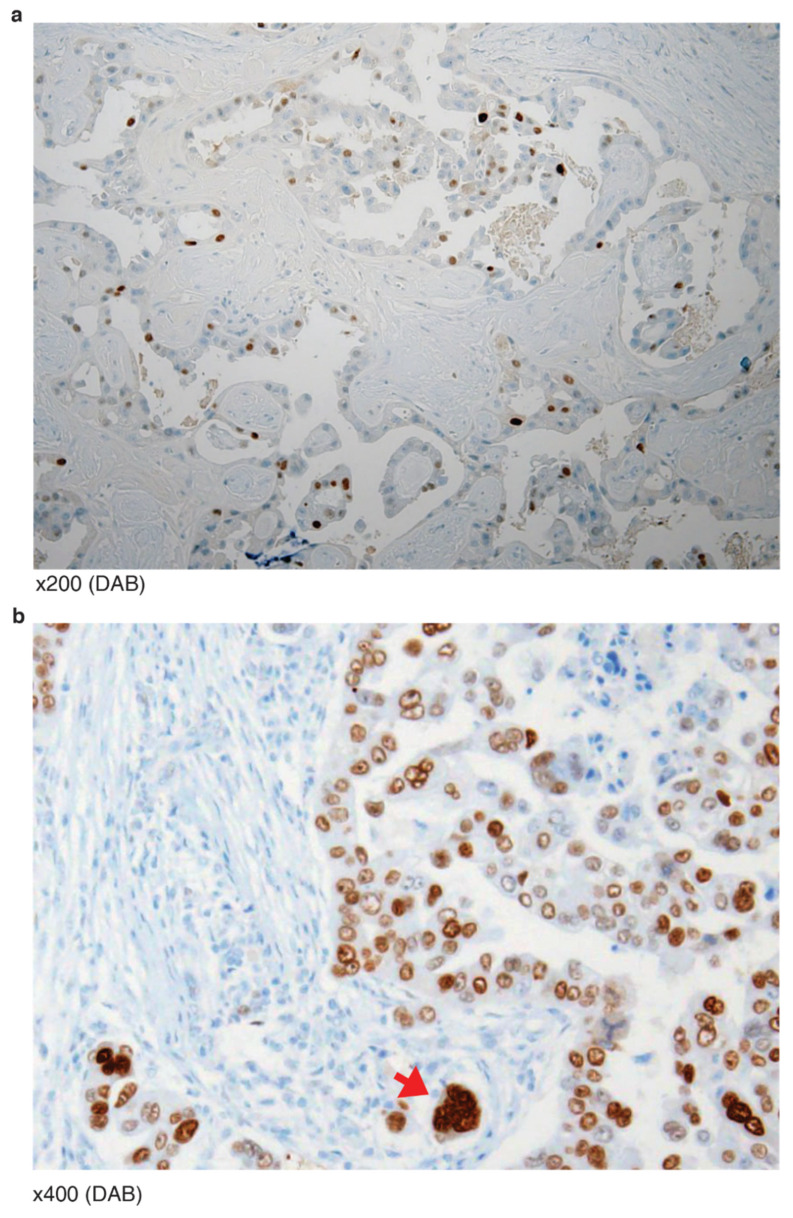
IHC mosaic patterns of p53 from two tumors of *TP53* NLS mutation (P301Qfs*44(del): (**a**) Well-differentiated serous carcinoma with intermittent partly positive signals. (**b**) Scattered positive cells with relatively high intensity are bizarre multinucleated cells (red arrow) undergoing aneuploidy.

There were eight HGSCa ovarian tumors of *TP53* mutation occurring at its nuclear localizing site (NLS). Five were nonsense mutations (E294*, E298* and three R306) and three were frameshift deletions (K319R fs*26 and two P301Q fs*44). Two of the three R306 cases presented cytoplasmic staining, consistent with the role of NLS. One of the two P301Qfs*44(del) (VAF 27.3%) cases was histologically equivocal between hybrid features of LGSCa and HGSCa, and the p53 IHC showed a mosaic pattern, well-differentiated serous carcinoma with intermittent, partly positive signals (Figure 6a). The signal intensity was stronger than wild-type, but weaker than the classic “all” pattern. The scattered positive cells with relatively high intensity were bizarre multinucleated cells (red arrow) undergoing aneuploidy (Figure 6b). Furthermore, the other P301Qfs*44(del) (VAF 82.7%) showed an even stronger signal, equivocal of “all” pattern staining. The rest of the four cases showed typical “none” pattern stainings.

#### 3.2.4. Oligomerization Domain (AA 325–356)

There were eight HGSCa and one metastatic carcinoma with oligomerization domain (OD) mutation. The sequencing results of two HGSCa (R337C, E339K) and one metastatic ca (I332T fs*13) were concordant with their IHC stainings—“none” pattern. The rest of the six HGSCa presented with three R342* nonsense mutations, one R342E fs*3 insertion, and two E343 nonsense mutations. Interestingly, the six HGSCa cases all showed “all” staining patterns. One of the R342* cases did show modest cytoplasmic staining together with variable nuclear staining (Figure 5b).

### 3.3. Typical Mosaic Patterns of p53 IHC in Mixed Carcinoma

**Figure 7:**
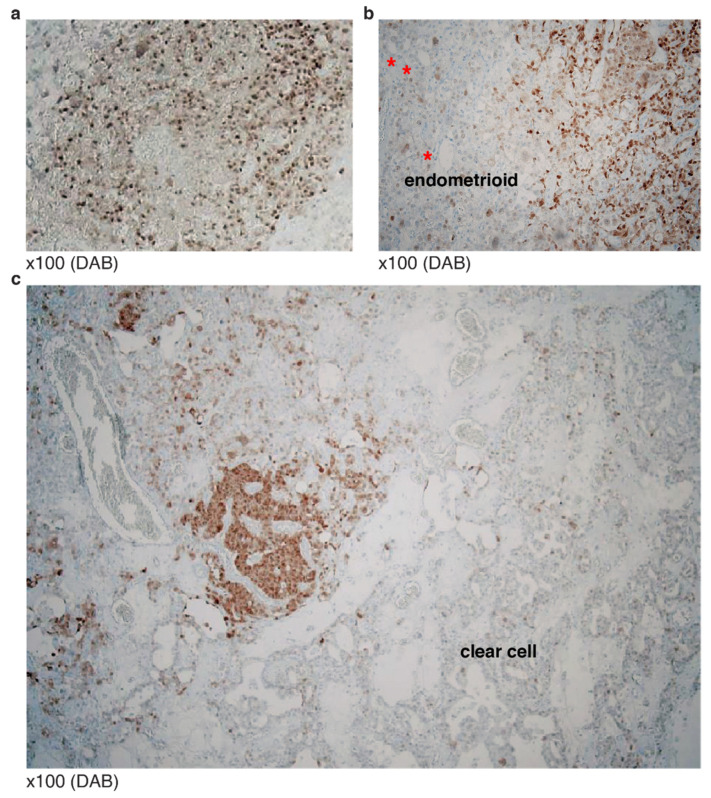
P53 mosaic staining patterns in mixed carcinoma: (**a**) Composite carcinoma including serous carcinoma; (**b**) endometrioid carcinoma with serous carcinoma, p53 poorly stained. Red asterisks: simple tubular structures often seen in endometrioid carcinoma; (**c**) mixed clear cell type with a WT pattern of p53, surrounding serous carcinoma showing diffuse staining.

Mosaic patterns of p53 IHC were seen in mixed epithelial composite carcinoma with TP53 missense or frameshift mutations. Histological heterogeneity of epithelial subtype in addition to serous carcinoma (Figure 7a), endometrioid (Figure 7b) or clear cell type was found (Figure 7c).

We report a full list of p53 IHC and NGS mismatching cases in Table 2.

## 4. Discussion

Missense mutation of *TP53* leads to protein conformational change, which results in prolonged turnover or stabilization, and accumulation in cells [17]. We performed a next-generation sequencing (NGS) panel including *TP53* and immunohistochemistry staining using a p53 antibody with an N-terminal epitope (at the transactivation domain) to detect the protein levels, subcellular locations, and additionally, the tumor mosaicism or subclonal heterogeneity. We report that the mutation types of the *TP53* gene in ovarian HGSCa generally matched the IHC results. *TP53* missense mutation (MM) mostly revealed diffuse and strong staining in nearly all tumor cells—the “all” pattern. Likewise, the *TP53* nonsense mutation (NM) showed perfectly negative staining in tumor cells—the “none” pattern. However, we also noted that there were about 10% mismatching cases—WT staining in MM or overexpression in NM.

p53 IHC could reliably predict TP53 mutations, with an overall level of accuracy as high as 91–97%, given that the optimized staining method and image interpretation were performed. Nevertheless, some HGSCa cases had detectable WT staining while harboring TP53 LOF mutations [18]. In this study, we were very careful to set the criteria of p53 IHC staining intensity. All the non-serous tumors with wild-type (WT) *TP53* gene and all normal samples showed a unanimously weak scattered staining pattern in no more than 30% of cells. Neither grouped positive areas nor strong staining nuclei were seen in any cases with the WT *TP53* gene. The cut-off criteria for p53 IHC staining varies across literature, mostly tumor type-dependent: there is a 5% cut-off criteria in hematologic malignancies [19]; 10% in brain tumors, esophageal squamous cell carcinoma and prostate adenocarcinoma [20,21,22,23]; 25% in oral squamous cell carcinoma [24], 40% to 50% in urothelial carcinoma [25,26], and 60% in ovarian carcinoma [27]. In our experience, all cases with the WT *TP53* gene manifested very weak signals with a scattered pattern, and never exceeded 30% of the total tumor cell population. Therefore, we used 30% as the cut-off value for WT staining. The plausible causes of discordant immunophenotypes and genotypes include limitation on mutant codon analysis, sample bias or heterogeneity or delayed p53 degradation by cellular stress [27].

The IHC-NGS mismatch frequencies varied across histological subtypes: 5–10% of HGSCa and around 30% of endometriod carcinoma and clear cell carcinoma. We repeated p53 IHC in two or more slides to minimize the limitation of representativeness. Additionally, we stained ER/PR and p16 for endometriod carcinoma, HNF1b for clear cell carcinoma, and WT1 for additive serous carcinoma, and performed a multiple rounding consensus triage. Tissue microarray (TMA) may encounter interpretation errors to fall in the “Pseudo-WT” trap in a mosaic pattern. To avoid such limitations, we used the whole mount slides instead of TMA. We first considered the intensity of p53, and secondly, the positive area distribution. We also analyzed the corresponding normal areas. Nevertheless, we found samples of positive staining exceeding 30% of the tumor cell population—either the mosaic or subclonal pattern.

Cytoplasmic stainings were fouind in around 2–3% of all serous carcinoma. All cytoplasmic staining harbored p53 mutations around NLD (305–322), NED (340–351), TD: 326–356 in the C-terminal. Dominant cytoplasmic staining with absent or variable weak nuclear staining has been accepted instead of aberrant nuclear staining, such as “strong nuclear staining with a blushing cytoplasmic pattern” [28]. Only two cases in the present eight cases with NLS mutation were compatible with strict criteria of cytoplasmic staining. We frequently experienced “all” patterned staining in *TP53* nonsense mutations at the oligomerization domain (OD). This is consistent with a recent report that nonsense and frameshift mutations occurring at the *TP53* carboxyl-terminus (exon 9–10) were frequently associated with high levels of p53 expression detected by IHC [29]. The OD is essential for DNA binding, protein–protein interactions, post-translational modifications, and p53 degradation. Interestingly, the phenotype of OD mutants often differs from that observed with classical DBD mutants [30]. Importantly, it is said that if the p53 protein cannot become an oligomer or a tetramer, its conformational change for DNA binding is less likely to occur either. However, even without tetramerization, active conformation changes and DNA binding still occur, so when the absolute amount of the p53 protein increases, its normal function can be compensated. Evolutionarily, proteins with suboptical status can supplement their functions by overexpression in cells, especially when they have feed-back transcriptional regulation. Therefore, the p53 OD nonsense mutant could have received feed-back transcriptional regulation. In addition, mutations in OD may cause less activity with protein degradation machinery, both of which increase the presence of protein.

Our study has some limitations, as well as some strengths. First, the use of additional p53 antibody targeting other protein domains would have helped understand the functional impacts of p53 mutations—early termination or splicing—in tumors. Secondly, our target panel did not assess intronic or promoter regions of the TP53 genes as well as loss of heterogeneity, which would help cases of TP53 nonsense mutations but not positive stainings. For an accurate diagnosis of ovary cancer, an error-free approach to HGSCa is most important. NGS is now popular for the p53 mutation type beyond p53 IHC analysis. With the provided solid criteria of p53 IHC interpretation together with NGS mutation typing, a perfect answer can be possible.

## 5. Conclusions

We reported the IHC of the p53 protein and NGS of mismatching *TP53* gene results from a series of ovarian HGSCa. We used the whole slide sections application to minimize the impact of intratumoral heterogeneity or staining bias. We found mismatching cases as well as mosaic patterns, which reflects subclonal expansions of HGSCa. The mismatching cases were enriched from the p53 c-terminal encoding nuclear localization domain and oligomerization domain, in which the subcellular locations as well as transcriptional feedback were affected. We conclude that for cases with TP53, and loss of function mutation of these domains, the IHC of the p53 protein is particularly informative.

## Figures and Tables

**Table 1:** Sample characteristics.

N Samples * (N DBD) ^†^	MM	NM	Frameshift		In-Frame		SP	WT	Total
			**Del**	**Ins**	**Del**	**Ins**			
HGSCa	130 (128)	32 (20)	27 (19)	6 (5)	3 (3)		4 (0)		202 (175)
LGSCa	1 (1)								1 (1)
Carcino-sarcoma	7 (7)	2 (1)							9 (8)
Endometrioid carcinoma	4 (4)		3 (1)			1 (1)		1 (0)	9 (6)
Mucinous carcinoma	5 (5)	1 (1)	2 (1)						8 (7)
Clear cell carcinoma	3 (3)								3 (3)
Metastatic carcinoma	3 (3)		1 (0)						4 (3)
Total	153 (151)	35 (22)	33 (21)	6 (5)	3 (3)	1 (1)	4 (0)	1 (0)	236 (203)

* MM = missense mutation; NM = nonsense mutation; SP = splicing; WT = wild type. ^†^ *n* of mutation at DNA binding domain (DBD).

**Table 2:** Cases of IHC and *TP53* mutation type mismatching (order by AA sequence).

Type	AA Change	Domain	Mutation Type	IHC Staining	Tumor %	VAF
endometrioid	V73W fs*50	TD	FSD	WT	30	10.3
clear cell	G105R	DBD	MM	WT	80	49.88
HGSCa	S183*	DBD	NM	“All”	70	42.67
HGSCa	F212_H214del	DBD	IFD	“All”	70	13.62
endometrioid	S261_G262dup	DBD	IFI	WT	40	16.71
HGSCa	G262del	DBD	IFD	“All”	80	32.92
HGSCa	R273H	DBD	MM	“None”	90	0.75
HGSCa	L289F fs*57	DBD	FSI	Cyto	70	23.66
HGSCa	P301Q fs*44	NLS	FSD	WT	60	27.28
HGSCa	P301Q fs*44	NLS	FSD	“All”	90	82.71
HGSCa	R306*	NLS	NM	Cyto	80	0.86
HGSCa	R306*	NLS	NM	WT + Cyto	80	0.60
HGSCa	R342E fs*3	OD	FSI	“All”	30	36.69
HGSCa	R342*	OD	NM	“All” + Cyto	90	37.4
HGSCa	R342*	OD	NM	“All”	50	7.08
HGSCa	R342*	OD	NM	“All”	60	44.4
HGSCa	E343*	OD	NM	WT	90	0.31
HGSCa	E346*	OD	NM	“All”	90	0.35

## Data Availability

All data available upon request.
